# Moniletherix

**DOI:** 10.11604/pamj.2013.15.53.2719

**Published:** 2013-06-12

**Authors:** Reza Yaghoobi, Amir Feily

**Affiliations:** 1 Department of Dermatology, Jundishapur University of Medical Sciences, Ahvaz, Iran; 2 Jahrom University of Medical Sciences, Department of Dermatology, Jahrom, Iran

**Keywords:** Monilethrix, hair, hair loss

## Images in medicine

Monilethrix is an autosomal dominant hair shaft disorder characterized by intermittent constrictions result in short and fragility hair. We present here two afghan siblings girl, 5 and 3 years, born of consanguineous marriage, come to our department of dermatology with complains of hair loss and inability to growth long hair of the scalp since birth. When the hairs reached a certain length, they were broking. There was no relevant familial history. On examination, we found diffuse thinning of scalp hair with broken, short and spars hairs. The length of hairs was less than 2 centimeter. Multiple keratotic papules were present on entire the scalp. Hairs in eyebrows also were involved. Diffuse hair loss seen in trunk and extremity. Nails and dental were normal appearance. Other systemic examination of hair showed beaded appearance with certain interval. Histopathologic examination of scalp showed misdirection with abnormal and tortuous appearance and bulb shaped shaft in some foci considering for monilethrix. Its differential diagnosis include monilethrix-like congenital hypothericosis, alopecia areata with variable activity in the course of disease, primary cicatricial alopecia (border of fibrotic area), chemotherapy induced alopecia, monilethrix-like effect from hair styling gel and pseudo-monilethrix. There is no specific treatment of monilethrix. Improvement by hormonal treatment reported in one study that hair growths increased after first menstrual period, so it suggested hormonal influence may improve hair growths. In literatures many different options for treatment of monilethrix proposed such as oral retinoid Griseofulvin and recently topical minoxidil 2%. One study showed improvement molinethrix after treatment by iron supplementation in iron deficiency anemia.

**Figure 1:**
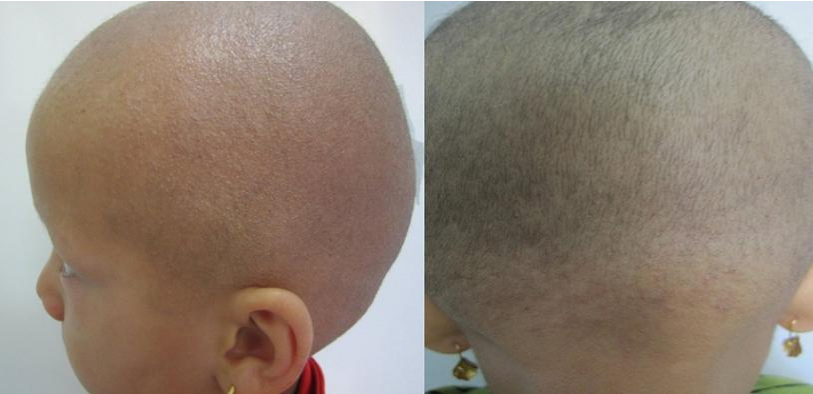
Two afghan sibling girl with hair loss and inability to growth long hair of the scalp since birth

